# Pregnancy protects the kidney from acute ischemic injury

**DOI:** 10.1038/s41598-018-32801-8

**Published:** 2018-09-28

**Authors:** Vasily A. Popkov, Nadezda V. Andrianova, Vasily N. Manskikh, Denis N. Silachev, Irina B. Pevzner, Ljubava D. Zorova, Gennady T. Sukhikh, Egor Y. Plotnikov, Dmitry B. Zorov

**Affiliations:** 1 Belozersky Institute of Physico-Chemical Biology, Lomonosov Moscow State University, Moscow, Russia; 2 Faculty of Bioengineering and Bioinformatics, Lomonosov Moscow State University, Moscow, Russia; 3 V.I. Kulakov National Medical Research Center of Obstetrics, Gynecology and Perinatology, Moscow, Russia; 4 Institute of Molecular Medicine, Sechenov First Moscow State Medical University, Moscow, Russia

## Abstract

A complex analysis of acute kidney injury (AKI) in pregnant women shows that it is caused by the interaction of gestation-associated pathologies and beneficial signaling pathways activated by pregnancy. Studies report an increase in the regeneration of some organs during pregnancy. However, the kidney response to the injury during pregnancy has not been addressed. We investigated the mechanisms of the pregnancy influence on AKI. During pregnancy, the kidneys were shown to be more tolerant to AKI. Pregnant animals showed remarkable preservation of kidney functions after ischemia/reperfusion (I/R) indicated by the decrease of serum creatinine levels. The pregnant rats also demonstrated a significant decrease in kidney injury markers and an increase in protective markers. Two months after the I/R, group of pregnant animals had a decreased level of fibrosis in the kidney tissue. These effects are likely linked to increased cell proliferation after injury: using real-time cell proliferation monitoring we demonstrated that after ischemic injury, cells isolated from pregnant animal kidneys had higher proliferation potential vs. control animals; it was also supported by an increase of proliferation marker PCNA levels in kidneys of pregnant animals. We suggest that these effects are associated with hormonal changes in the maternal organism, since hormonal pseudopregnancy simulated effects of pregnancy.

## Introduction

Acute kidney injury (AKI) remains one of the major pathological factors in clinical practice worldwide. Although it is a great burden for modern society due to the high mortality rate from AKI which was estimated to be from 20 to 40%^1^, AKI in pregnancy may become at least two times more dangerous, since it threatens both mother and fetus. It stems from various obstetrics complications: prerenal (hemorrhage, hyperemesis gravidarum, congestive heart failure, sepsis), intrarenal (acute tubular necrosis, pyelonephritis, renal cortical necrosis, thrombotic microangiopathy, preeclampsia/HELLP syndrome, acute fatty liver of pregnancy, glomerulonephritis, medications), and postrenal (obstruction) causes^2^, which, if left untreated or poorly treated, can cause irreversible renal damage and ultimately can lead to severe, and even fatal, complications for both the mother and the baby. The obstetric AKI contributes significantly to the maternal morbidity. In Canada, it reached 2.9% of all fatality cases among pregnant women^3^. Overall, pregnancy is a major factor affecting kidney diseases that differ greatly among countries and populations and can occur during/after 10% of all pregnancies in developing countries. It forces us to urgently cope with the problem, complicated by the fact that the alleged targets of a potential protective exposure are at least two organisms, different in age and susceptibility.

Since the middle of the XX century, the incidence of AKI in pregnancy has been significantly reduced due to a successful fight with infections. However, at the beginning of the XXI century, rates have increased. In Canada and the United States, obstetric AKI increased from 1.6 and 2.3 per 10 000 deliveries in 2003 to 2.3 and 4.5 per 10 000 in 2007. In developing countries, the situation is worse and can reach 70 cases per 10 000 deliveries (Morocco). Though, for now, it has positive dynamics: for example, while in India in 1980 AKI was diagnosed in 15% of hospitalized pregnant patients, it dropped to 1.5% in 2010; similar numbers were reported for China^4^. Kidney injury during pregnancy is associated with comorbid conditions and outcomes^5^. At least two reasons can explain the losing battle against AKI in pregnancies in developed countries: 1) the loss of the efficiency of antibiotics forcing the use of drugs with serious nephrotoxic effects in pregnant women, and 2) a general trend of aging in the human population including pregnant women.

The complexity of considering AKI in pregnant women is caused by the interaction of two factors: negative gestation-associated pathologies and potentially beneficial signaling pathways activated by pregnancy. Indeed, studies report an increase in regeneration rates of some organs during pregnancy, which was reviewed by us elsewhere^6^. For example, pregnancy induces increased liver regeneration in old rats by four times and decreased post-surgery mortality 5-fold^7^. Similar effects were reported for muscles^8^ and spinal cord^9^. However, kidney response to injury during pregnancy has not yet been addressed.

An obvious contradiction emerges: clinical statistics and cases suggest that pregnancy is a major risk factor for kidney pathologies, but some fundamental studies suggest that it might have the opposite effect. These two superficially controversial points of view can both be true, since clinical studies operate with complex medical processes: different complications and side factors can affect outcome even more than the initial pathology, while fundamental studies address isolated and individual diseases and pathologies.

Thus, present work aims to address the AKI development during pregnancy directly: to test the impact of pregnancy on AKI severity and to elucidate specific mechanisms affecting kidney tolerance.

## Results

### Pregnancy effect on AKI

**Figure 1:**
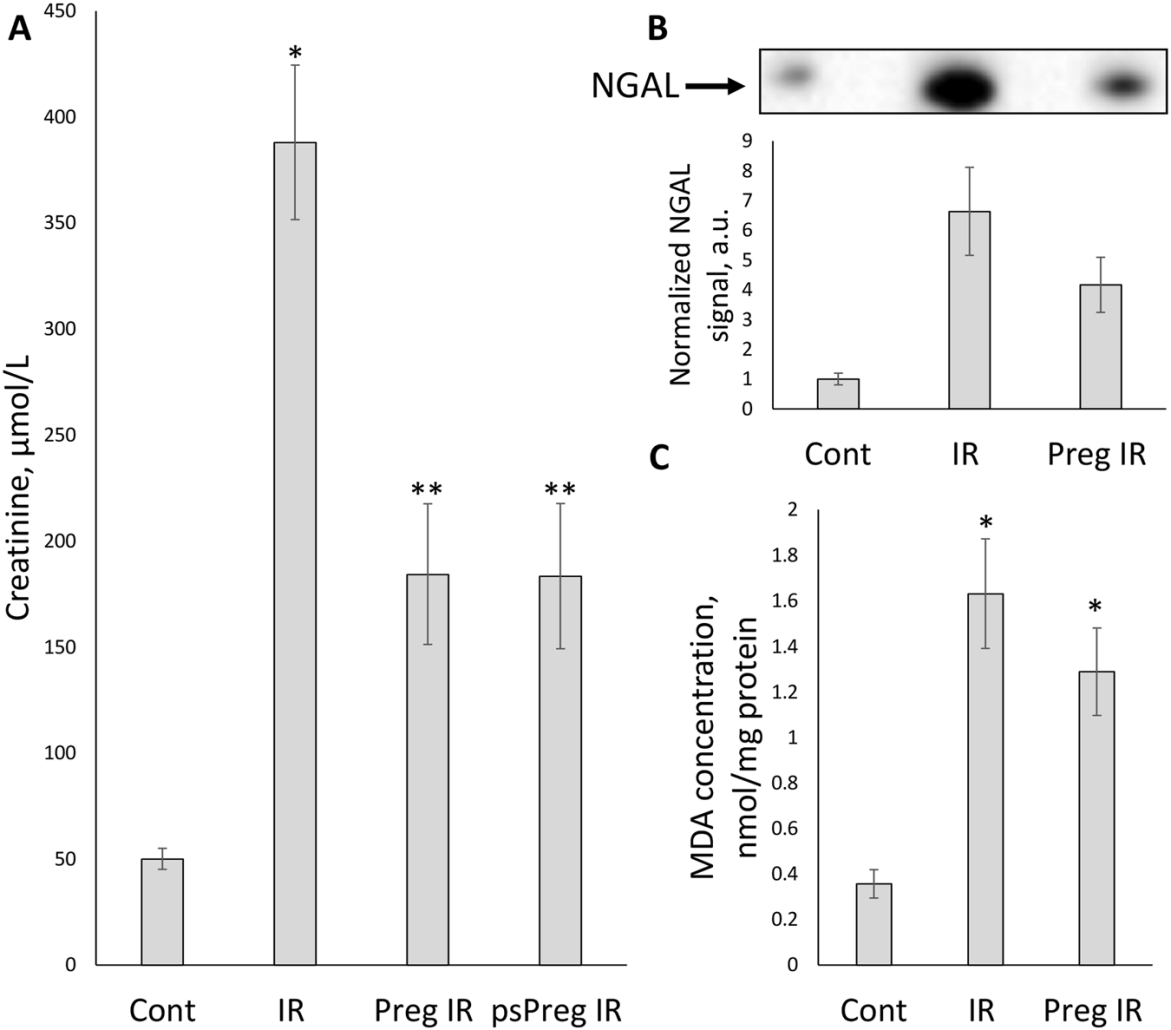
Renal dysfunction and oxidative damage 48 hours after kidney I/R. (**A**) Renal insufficiency indicated by an increase in serum creatinine levels (µmol/L) after I/R in non-pregnant (IR, n = 10), pregnant (Preg IR, n = 10) and pseudopregnant (psPreg IR, n = 8) rats, compared to control (Cont, n = 10). (**B**) AKI indicated by a concentration of a kidney-damage marker NGAL in serum of IR, Preg IR and Cont (n = 2; 6; 3, respectively). (**C**) Oxidative tissue damage indicated by the presence of a lipid peroxidation product (MDA, nmol/mg protein) in kidney tissue of IR, Preg IR and Cont (n = 5; 5; 4, respectively). *p < 0.01 compared to control group, **p < 0.01 compared to I/R group.

Since acute renal damage occurs during ischemia/reperfusion (I/R)^10^, this approach was used as a model of AKI in the study. One of the conventional clinical markers of renal insufficiency – serum creatinine level – was determined 48 hours after injury. Figure 1A shows that I/R dramatically increased serum creatinine level (up to 388 ± 36 µmol/L from 50 µmol/L in control animals), whereas pregnancy or pseudopregnancy demonstrated a remarkable protective effect decreasing serum creatinine concentration 2-fold (up to 184 ± 33 and 183 ± 34 µmol/L respectively) comparing to I/R group.

The marker of early renal damage, neutrophil gelatinase-associated lipocalin (NGAL), which was determined in serum also 48 hours after ischemic insult, demonstrated a 7-fold increase (Fig. 1B); the same parameter in pregnant animals dropped by 40%, similar to the creatinine level changes.

The appearance of the renal damage indicators in blood was associated with elevated levels of peroxidative lipid product, malondialdehyde (MDA) in renal tissue. This marker is a recognized reporter of oxidative tissue damage, which is known to occur during ischemia. 48 hours after injury, the levels of MDA rose 5-fold, while the same parameter in pregnant animals exposed to identical procedures was 20% lower (Fig. 1C). Thus, judging from the changes in the renal damage indicators, pregnancy alleviates the burden of I/R.

### Acute tubular necrosis (ATN)

**Figure 2:**
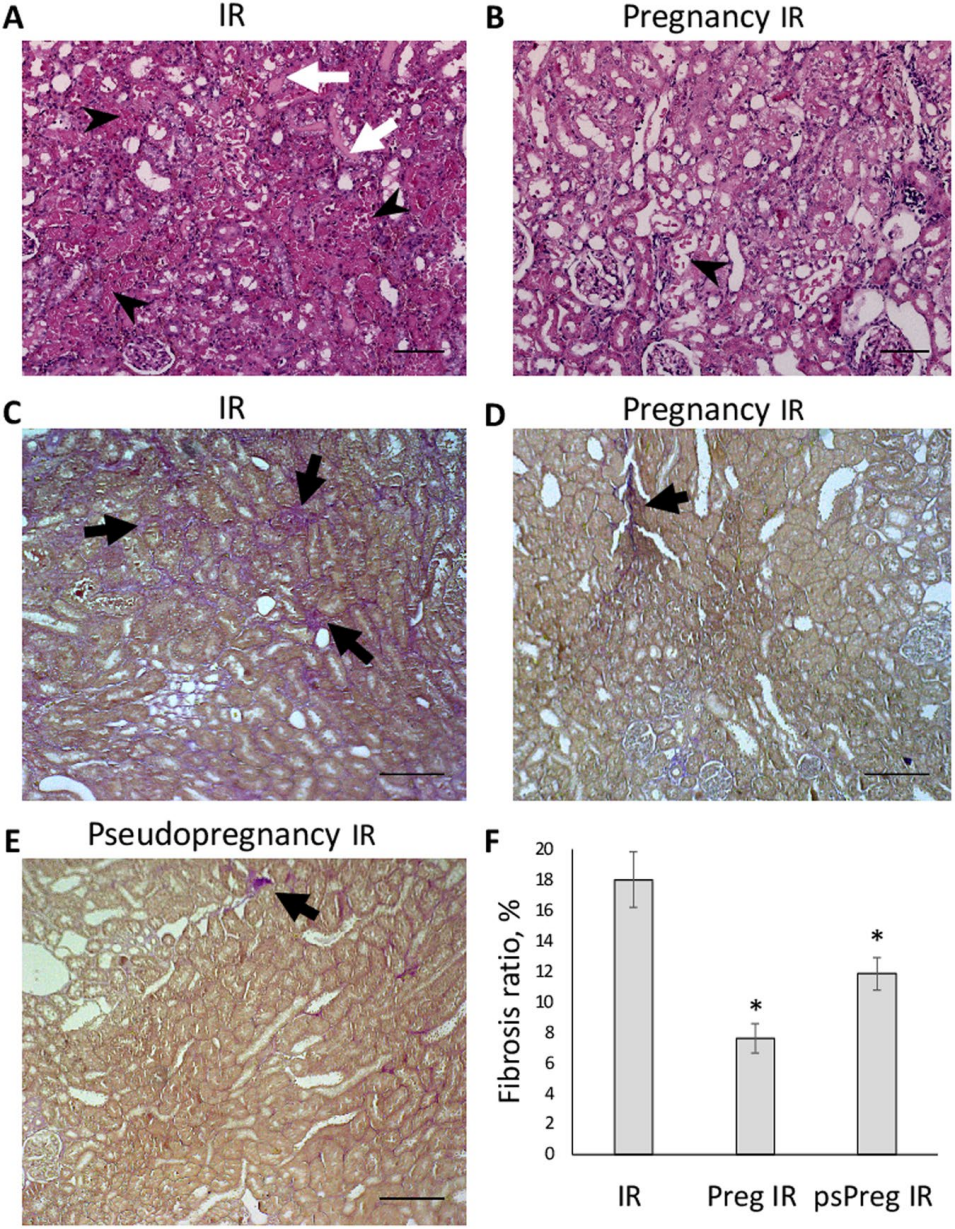
Histological assessment of rat kidneys 1 day and 2 months after I/R. (**A**,**B**) Effects of pregnancy on ischemia-induced pathological features in renal morphology. Kidney sections were stained with hematoxylin and eosin. (**A)** Kidney from a non-pregnant rat taken 1 day after 40-min ischemia. Tubular necrosis (shown by black arrowheads) and noticeable cast formation (white arrows) are seen in kidney tubules. (**B**) Kidney from a pregnant rat taken 1 day after I/R. (**C**–**E**) Representative histological images demonstrating fibrosis in the rat kidney cortex 2 months after ischemia in non-pregnant (IR), pregnant (Pregnancy IR) and pseudopregnant (Pseudopregnancy IR) rats. Significantly more fibrosis lesions are seen as purple-stained tissue structures (black arrows) in I/R group, comparing with two others. Bars, 100 μm. (**F**) Calculated fibrosis score in kidneys of non-pregnant, pregnant and pseudopregnant rats (number of slices n = 17, 18, 14, respectively). Note significantly lower fibrosis ratio in groups of pregnant and pseudopregnant comparing to non-pregnant animals. *p < 0.01 compared to I/R group.

Effect of pregnancy on ATN was examined one day after ischemic injury exploring histopathological changes in kidneys. In I/R-exposed kidneys from non-pregnant female rats, a pronounced ATN in renal cortex was observed (Fig. 2A) with ATN score = 3 (using the scale: 0 – no changes; 1 – mild; 2 – moderate, 3 – severe changes). Necrotic tubules occupied large areas of cortex tissue; moreover, many tubules demonstrated desquamated or degenerated epithelial cells. In addition, multiple hyaline casts in tubular lumen could be seen (score = 3), which indicates possible obstruction of downstream nephron segments and compromised primary urine flow. Pregnancy resulted in lower histopathological scores in rats that underwent kidney ischemia. In pregnant animals after I/R, only few focal necrotic loci could be observed, while most of the kidney tissue structure was preserved (Fig. 2B) with ATN score = 1. In these animals, hyaline casts were seldom (score = 1), which indicates a lesser degree of downstream nephron obstruction. These observations suggest that acute tubular necrosis after I/R injury is significantly less severe in the group of pregnant animals.

### Fibrosis

One more marker of cell damage is the fibrotic transformation of the tissue, which is not only an indicator of the past pathogenic stimulus but also a strong factor that interferes with the normal functioning of the tissue and very often becomes the cause of organ failure. It has been demonstrated that a single episode of renal I/R leads to chronic kidney fibrosis and dysfunction^11,12^, and it is a noteworthy that the detailed mechanisms of I/R-mediated kidney fibrosis are poorly understood. In our experimental model, fibrosis level was evaluated two months after initial injury using a histological approach. Figure 2(C–F) shows that the fibrosis score in kidney cortex was significantly higher in a group of non-pregnant animals than in groups of pregnant or pseudopregnant rats. Pseudopregnancy showed a smaller protective effect than actual pregnancy reducing the fibrosis only 1.5 times, compared to 2.3 times of pregnancy.

Thus, we show that pregnancy, both natural and hormone-simulated, partially protects against fibrosis induced by rat kidney I/R.

### Oxygen-glucose deprivation effect on primary kidney tubular epithelial cell culture

**Figure 3:**
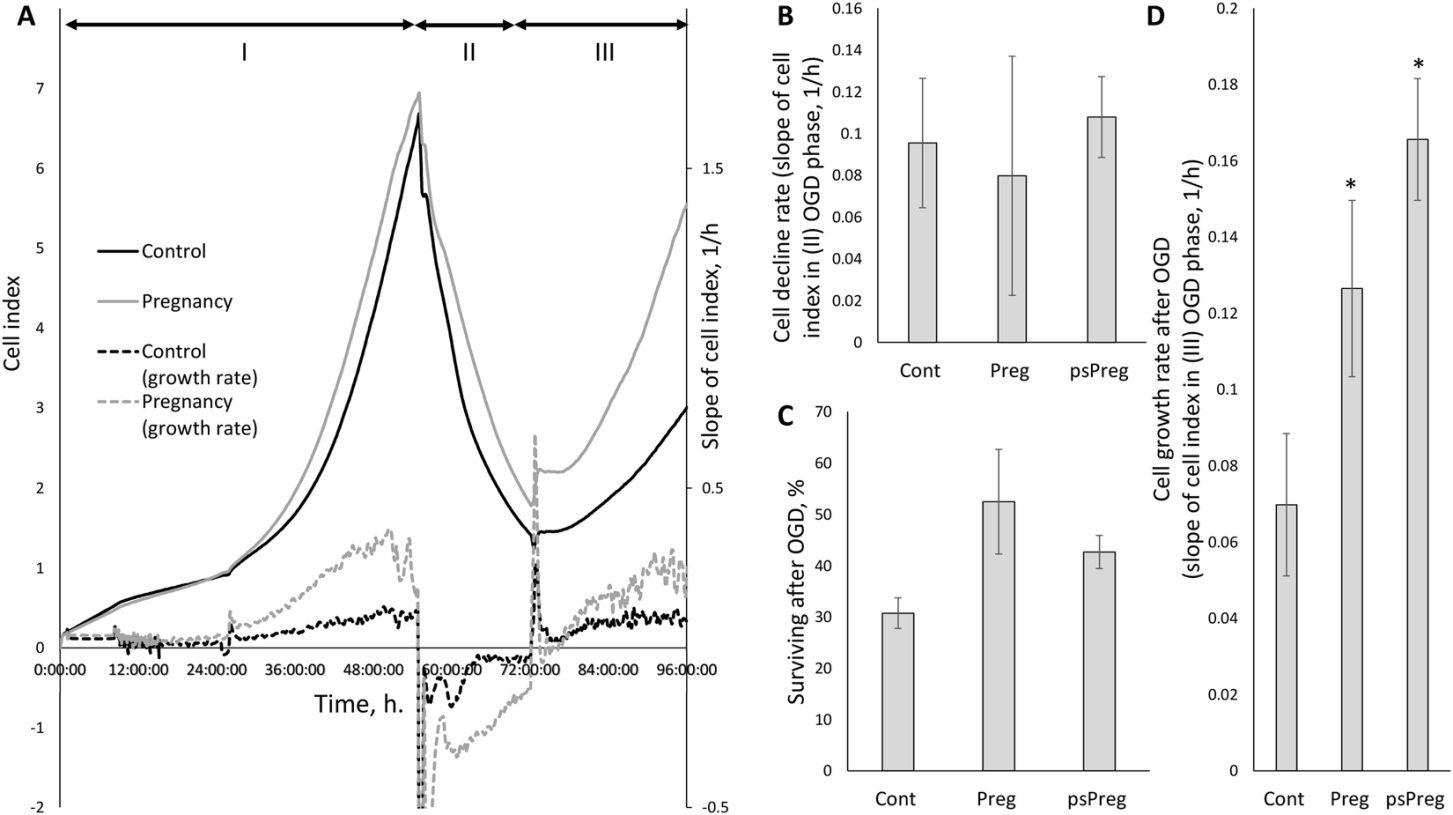
Primary kidney tubular epithelial cell culture viability after ischemia/reoxygenation *in situ*. (**A**) Real-time proliferation and growth rate of cells obtained from pregnant and control animals: I, cell growth under normoxic conditions; II, OGD phase; III, phase of restoration of oxygen and glucose supply. Solid lines relate to cell index curves, dashed lines indicate growth rate (derivative of cell index curve). (**B**) Cell death *during* OGD. (**C**) Survival *after* OGD, and (**D**) growth rate measured by iCELLigence after OGD of cells from control (Cont), pregnant (Preg) and pseudopregnant (psPreg) rats (n = 8; 4; 3, respectively). *p < 0.05 compared to control group.

**Figure 4:**
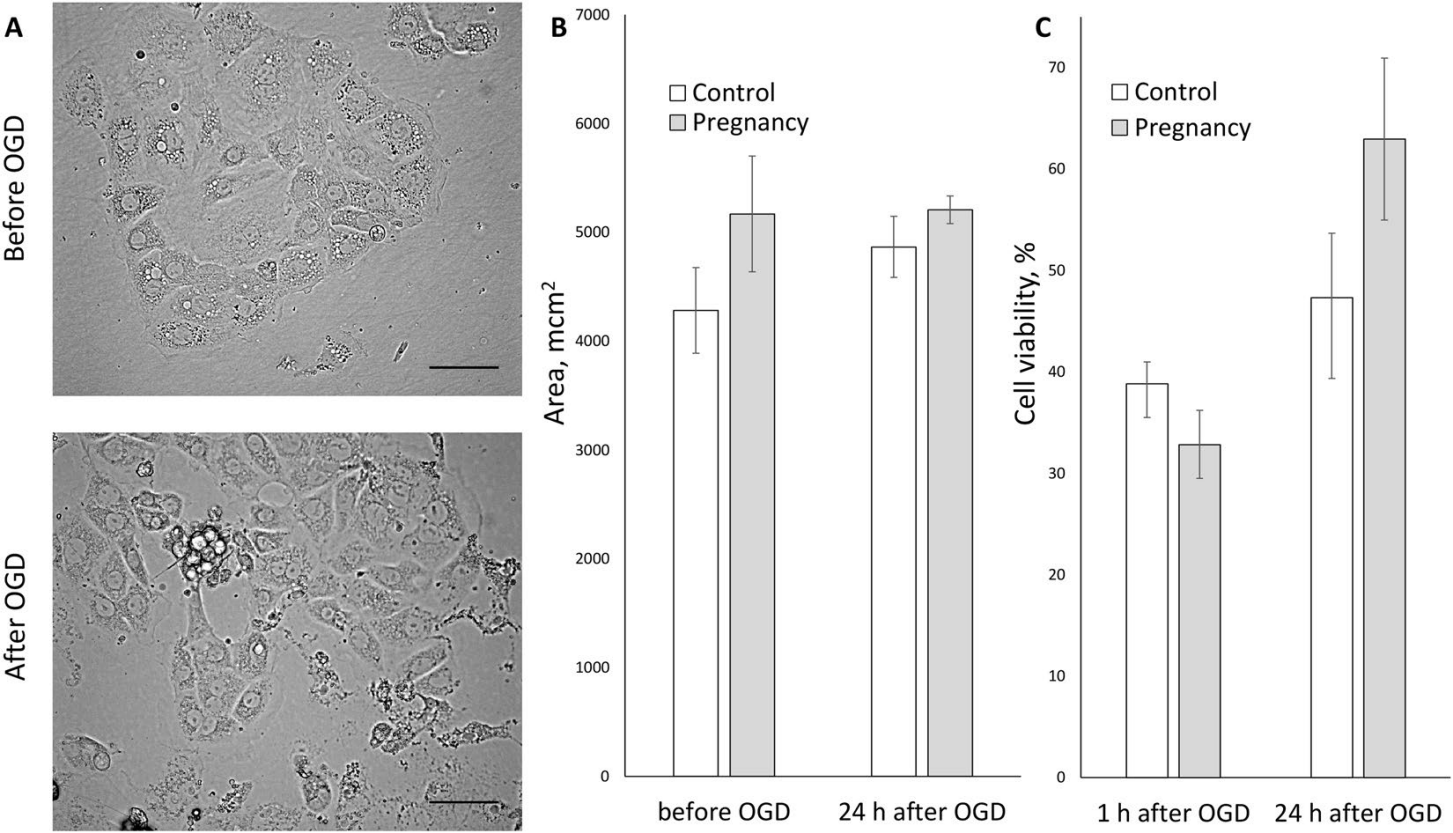
Primary kidney tubular epithelial cells viability and area after OGD. (**A**) Phase contrast microscopy of primary kidney tubular epithelial cells culture from adult control rat before and after OGD. Bars, 100 μm. (**B**) Kidney tubular epithelial cells area before and 24 hours after OGD. (**C**) Cell viability estimated by MTT test 1 and 24 hours after OGD.

One of the conventionally used models to simulate ischemia *in situ* is the deprivation of cultured cells by glucose and oxygen (OGD)^13^. Taking OGD as an ischemic model, we addressed renal cells response to ischemic injury of primary kidney tubular epithelial cell culture from adult rats by using a real-time cell analysis system iCELLigence (Fig. 3A). We found that neither growth rate of epithelial cells obtained from pregnant or pseudopregnant animals differs from control group before OGD, nor all groups differ in the cell death rate during OGD (Fig. 3B) and in their survival after OGD as well (Fig. 3C). Although there was only a trend for the increased viability of cells of pregnant and pseudopregnant animals, a statistically significant (p-value ≤ 0.05) 2-fold increase in cell growth rate after OGD was obvious (Fig. 3D). We have also addressed if these results can be explained by cell hypertrophy through measurement of cell areas before and after OGD. We did not observe any significant changes in cell size between groups (Fig. 4A,B), which indicates the absence of profound hypertrophy.

Similar results were obtained using a standard methyl thiazol tetrazolium (MTT) cell viability test. There was no difference in cells viability measured 1 hour after OGD between control group and groups of pregnant animals (Fig. 4C), while MTT test 24 hours after OGD showed that cells obtained from pregnant animals demonstrated 15–20% increased viability, comparing to cells obtained from control animals (Fig. 4C).

### Pregnancy effects on kidney regeneration

The issue of rehabilitation after injury remains one of the important subjects and purposes of the research. The ability to recover lost functions is largely determined both by the rate of elimination of damaged structures (in particular, through the process of autophagy^14^), mobilization of resident stem cells^15,16^ and by the activation of signaling regenerative cascades in remaining differentiated cells^17,18^. Proliferation factors have been shown to play an important role in the latter processes, many of which are attributed to rejuvenating functions^19–22^.

**Figure 5:**
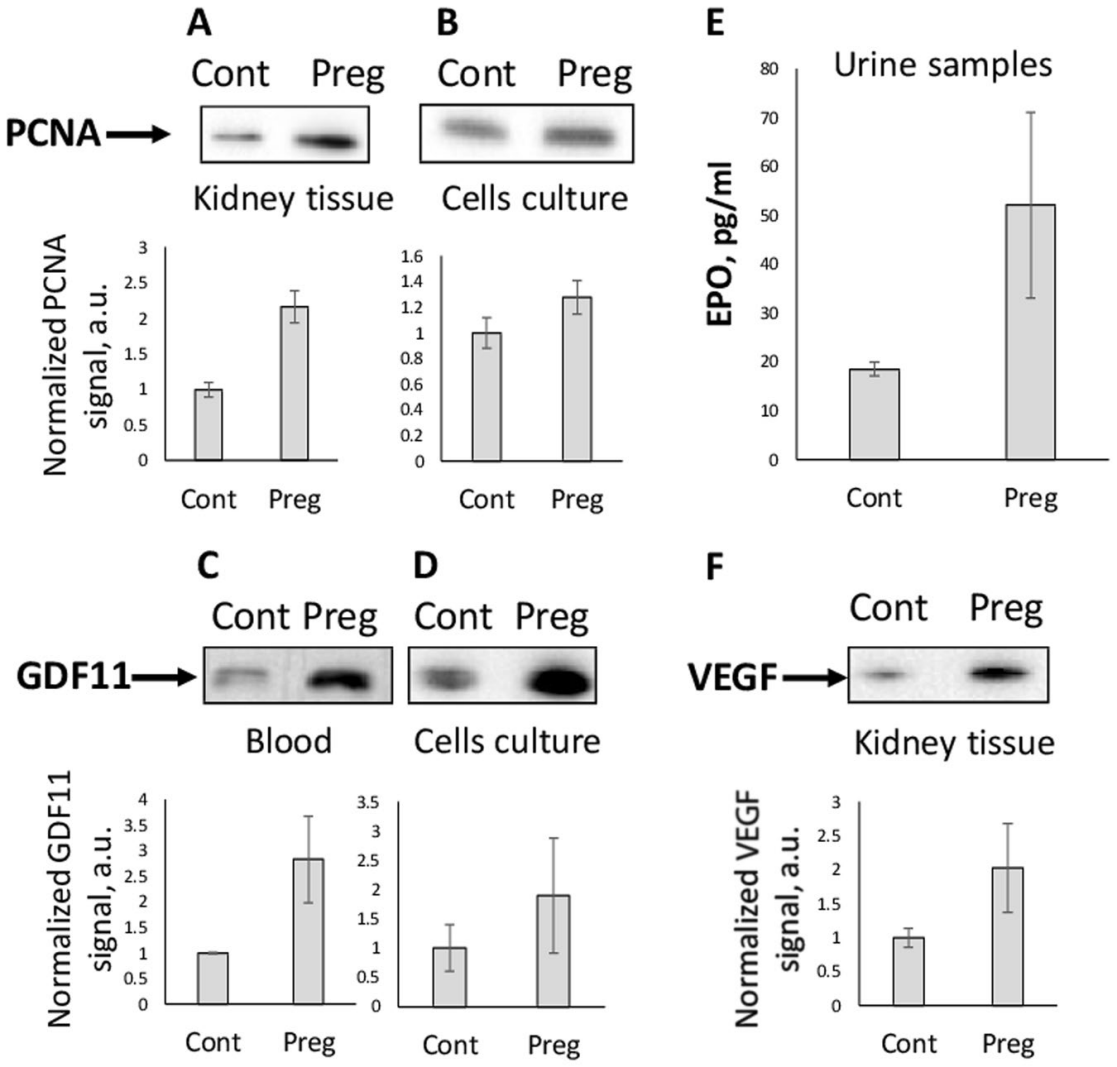
Regeneration markers levels in kidney and blood. Levels of (**A**,**B**) proliferation marker PCNA, (**C**,**D**) rejuvenation marker GDF11, (**E**) erythropoietin (EPO) level in urine estimated by ELISA, (**F**) angiogenesis factor VEGF in kidney tissue, serum and primary kidney tubular epithelial cells from control (Cont) and pregnant (Preg) animals estimated by Western blotting. Note that pregnancy is associated with the increase of all of these factors.

In this study, we found that levels of proteins associated with cell proliferation and regeneration were increased in pregnant animals. The levels of proliferating cell nuclear antigen (PCNA), a well-known proliferation marker, were doubled in pregnant rats kidney tissue comparing to control (Fig. 5A). The same trend was observed in PCNA levels in primary kidney tubular epithelial cell culture, though to a lesser extent (Fig. 5B). A similar tendency was seen for the rejuvenation marker growth differentiation factor 11 (GDF11). In serum and cultured kidney cells, GDF11 levels were noticeably higher in the group of pregnant animals (Fig. 5C,D). A two-fold increase in the content of vascular endothelium growth factor (VEGF) was also observed in kidney tissue (Fig. 5F) and a 3-fold increase of erythropoietin concentration measured in urine from pregnant animals (Fig. 5E).

### Pregnancy effects on ischemia tolerance signaling

**Figure 6:**
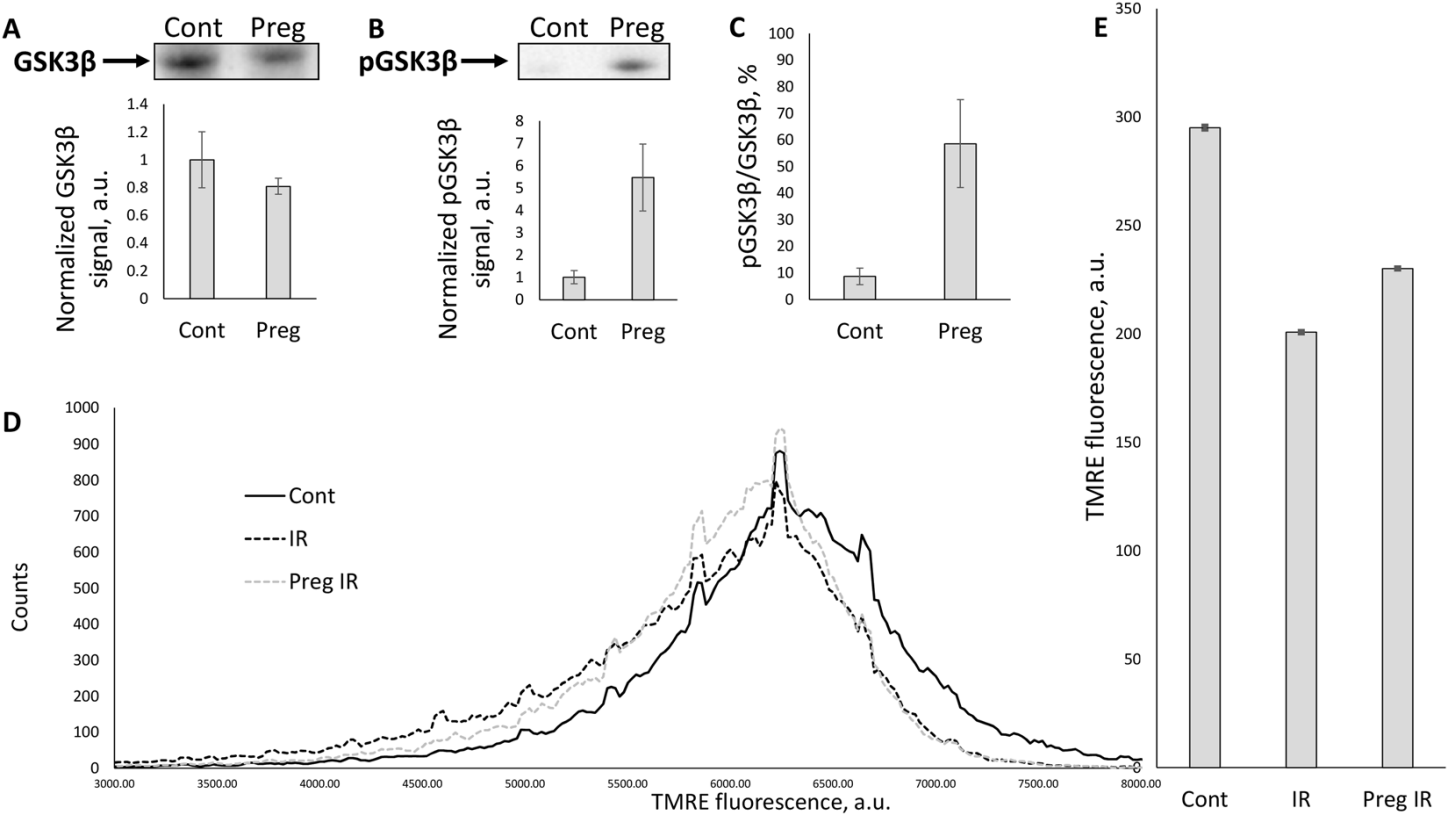
Pregnancy effects on ischemic tolerance signaling and mitochondria functioning. The decrease in levels of GSK3β (**A**) and increase in levels of pGSK3β (**B**) thus significantly increasing pGSK3β/GSK3β ratio (**C**) in kidney tubular epithelial cell culture from pregnant (Preg) compared to control (Cont) animals. (**D**) Mitochondrial transmembrane potential measured by flow cytometry of TMRE-loaded kidney mitochondria, isolated from intact non-pregnant animals (Cont), non-pregnant (IR) and pregnant animals (Preg IR) after I/R. (**E**) Note that I/R causes the decrease in mean TMRE fluorescence intensity in non-pregnant animals indicating a drop of mitochondrial membrane potential, while in a group of pregnant rats such mitochondrial dysfunction was less pronounced.

In protective signaling pathways, glycogen synthase kinase 3β (GSK3β) plays a key role: levels of phosphorylated GSK3β are highly associated with prognosis for better survival^23–25^. In our experiments, GSK3β in the non-phosphorylated (enzymatically active) form, which is described to contribute to a number of cellular damaging processes^24,26^, was shown to be underexpressed in primary kidney tubular epithelial cell culture from pregnant animals (Fig. 6A). On the other hand, the inactive (Ser-9 phosphorylated) form of GSK3β (pGSK3β) was much more abundant in kidneys of pregnant rats (Fig. 6B). This means that in kidneys of pregnant animals the ratio pGSK3β/GSK3β was 6–7 times higher than in the same tissue of non-pregnant animals (Fig. 6C).

In addition to that, mitochondria isolated from control kidney tissue accumulate significantly more mitochondrial membrane potential probe TMRE than mitochondria from kidneys (Fig. 6D) injured by I/R that points to lower transmembrane potential and mitochondrial damage after I/R. Pregnancy, however, partially prevented the alterations caused by I/R: it can be noted, that left tail of TMRE-fluorescence intensity distribution (showing the presence of low potential-mitochondria) in pregnant I/R group is shorter than in non-pregnant I/R group (Fig. 6, D and E), which indicates, that the population of mitochondria with low transmembrane potential was smaller in the group of pregnant animals. Thus, in the group of pregnant rats, functional heterogeneity of renal mitochondria seems to be lower than in a group of non-pregnant animals.

### Possible role of fetus in afforded protection

Apart from hormonal changes, enhanced tolerance to I/R damage in pregnancy could be provided by some fetal factors. Fetus-derived protection can include two different issues: it can occur due to the effect of some fetal regulatory factors, or it can be derived from the direct impact of fetal stem cells. The latter phenomenon coined “microchimerism” is quite well established: in different species, it has been shown that stem cells from the fetus circulate in the maternal bloodstream and can be found in various tissues where they can differentiate^27–33^. We found that cells carrying Y-chromosome (i.e., originating from male fetuses) can be detected in mothers renal tissue (Suppl. 1A). In addition, we crossbred wild-type female mice with GFP-transgenic males, which resulted in some embryos being GFP-transgenic (Suppl. 1B). When analyzed these pregnant mouse kidneys, we detected solitary GFP-fluorescent cells, obviously, originated from GFP-transgenic fetus in mother renal tissue (Suppl. 1С).

## Discussion

The impact of pregnancy on the course of normal and pathological processes in the organism has been described unsystematically, although there is a general, unconfirmed opinion about the rejuvenating effect of pregnancy. In our recent analysis^6^, we have summarized a data set, concluding that in some cases the consequences of pathological processes in pregnancy are mitigated. On the other hand, we noticed that for carriers of severe pathologies, pregnancy could aggravate the course of the pathological process.

In this study, we tried to fill a gap in the data on the impact of pregnancy on the pathological processes in the kidney. As to the degree of damage to the kidney, we used classic indicators that appear in the blood as a result of kidney damage, for example, creatinine, a product of creatine decomposition, which functions primarily in muscle tissue and is subject for removal through the kidneys. Its elevated blood levels indicate an impairment of the excretory function of the kidney. For strengthening the data on the occurrence of ischemia-associated AKI, we used another indicator NGAL, which has been recently introduced in clinical practice and is considered to be a more sensitive marker reflecting the earliest stage of kidney damage when the response from the other indicators is minimal. We also used conventional histopathological approach to diagnose the acute kidney tissue damage directly. The evaluation of the AKI using different markers of renal damage is critical, since AKI belongs to syndromes with varied clinical manifestations from a small loss of glomerular filtration rate to the entire organ failure^34^.

The primary goal of this work was to study the severity of AKI during pregnancy. We demonstrate that AKI caused by I/R is significantly less severe in pregnant animals than in the control group (Fig. 1). Indeed, using creatinine as an index of AKI, we found more than a 2-fold drop in its blood levels in the pregnant animal scomparing to non-pregnant after AKI, which indicates that renal function is significantly less impaired after injury in pregnant animals. Serum NGAL levels after I/R are also higher, but in the pregnant I/R experimental group its levels are lower, i.e., similar to the behavior of creatinine levels.

The renal excretory impairments were associated with oxidative stress indexed by an increased lipid peroxidation using measurements of MDA levels in the serum. Similar to the behavior of creatinine and NGAL, we detected lower levels of the MDA in renal tissue of pregnant animals exposed to I/R than in that of non-pregnant. This fact indicates that during pregnancy lipids were less oxidized possibly due to lower levels of I/R-induced oxidative stress.

Histopathological examination revealed that ATN was less severe in pregnant animals one day after injury, which directly proves that kidney tissue, was more tolerant to ischemic damage. Moreover, we observed not only a lower level of ATN, which is an indicator of tissue damage, but also increased levels of hyaline casts in tubular lumen that was associated with impaired kidney functions due to obstruction, and these changes were much less pronounced in pregnant animals.

This data demonstrates that pregnancy protects kidney both from dysfunction (indicated by serum creatinine levels) and acute injury (as judged by histopathological features, NGAL and MDA levels) caused by ischemia.

The remote pathological consequences of organ damage are associated with significant loss of functional cells (e.g., cardiac myocytes in heart, neurons or astroglial cells in the brain and excretory epithelial cells in kidney). Ultimately, instead of these cells, the organ acquires cells forming a fibrotic tissue. As a result of such substitution, normal organ functioning is impaired: from a slight violation of the organ functioning including impairment of tissue regeneration and eventually leading to chronic kidney disease and end-stage kidney disease terminating with organ failure. Such a role of damage-induced fibrosis is a subject of concern being a focus of numerous studies. We evaluated the development of renal fibrosis two months after initial damage and demonstrated that pregnant animals exposed to ischemic renal damage have a remarkable lower level of fibrosis in the kidney. This fact can have several explanations. The initial damage was less severe, i.e., the organ was more tolerant to the damage; the following secondary inflammatory damage was less severe (and the fact that anti-inflammatory effect of progesterone as shown to have some protective effect in pregnancy^35,36^ confirms it); the progression of fibrosis is somehow retarded mostly by modulation of fibrogenic factors^37–41^. Alternatively, the tissue becomes more prone to regeneration^42,43^. No doubt, these mechanisms can operate simultaneously in the kidney ultimately affording protection.

To unravel the details of pregnancy-related protective mechanisms, we turned to the cellular level using primary kidney tubular epithelial cell culture from adult rats. It is worth noting that unlike embryonic kidney cell cultures, which are widely used, kidney cells from adult animals are very difficult to cultivate, and they are used rarely. Embryonic cells did not meet our goals for obvious reasons: embryonic cell culture cannot be affected by pregnancy in the normal way. Also, it is possible that embryonic cell cultures could naturally acquire the same protective mechanisms as renal cells from pregnant animals, either due to their young age-associated properties or through exchange with the maternal bloodstream. As to elucidation of a possible enhanced proliferation in pregnancy, using the OGD model as an *in vitro* model of ischemic injury, cell proliferation was monitored in real-time covering different phases of OGD exposure. Neither significant difference in cell proliferation *before* OGD was observed, nor any serious differences were found at the very moment of OGD: cell decline rate did not differ significantly, and thus no significant difference in cell survival was observed as well. The last parameter was also confirmed using a standard cell viability MTT assay collectively yielding the same result. On the other hand, cell cultures response *after* OGD was different: cultural cells obtained from pregnant animals proliferated at the significantly higher rate after OGD, and MTT test 24 hours after OGD showed a remarkable increase in cells viability.

In an attempt to describe the renal protective mechanism in pregnancy, we suggested that kidney cells derived from pregnant animals might not be more protected from damage than non-pregnant animals’ cells, but they recuperated faster after damage and were more prone to recover from tissue and cellular damage by more active proliferation. The latter suggestion is supported not only by cell proliferation data from real-time monitoring, but also by analyzing data on the levels of proliferation factor PCNA that was measured both in kidney tissue and in cell culture, and in both cases its levels were higher in the group of pregnant animals. Also, the blood levels of GDF11, one of the most intriguing regeneration and rejuvenation factors^19,21^, were also higher in blood and cells obtained from pregnant females. This factor plays an important role in “rejuvenation” proven in the model of heterochronic parabiosis made through the surgical connection of bloodstreams of young and old animals leading to the conversion of animals with old phenotype to young phenotype based on the restoration of regenerative capacities^44^. It is tentative to consider pregnancy as a natural form of parabiosis: indeed, a mature adult animal (mother) is at least partially connected by the bloodstream to a juvenile one (fetus).

We proceeded the analysis of several additional signaling pathways and factors activated in pregnancy, namely VEGF and erythropoietin. VEGF is a downstream effector of the relaxin-related signaling pathway particularly linked to angiogenesis^45–47^ and its levels in kidney tissue of pregnant animals were found to be two times higher than in non-pregnant. Levels of erythropoietin, another signaling molecule, were also elevated during pregnancy. Erythropoietin is secreted in kidneys, and apart from its primary regulation of erythropoiesis, it can carry anti-ischemic, anti-apoptotic and angiogenesis functions^48–50^. Ultimately, our findings suggest that pregnancy-associated changes upregulate regeneration- and proliferation-associated pathways. All named factors, directly and indirectly, belong to cell-protective signaling pathways ultimately affording higher protection to the maternal organism.

As to the role of the fetus in afforded protection against ischemia, our data demonstrate that unidirectional talk between fetus and mother does exist which is supported by revealed microchimerism expressed in fetus cells harboring mother’s renal tissue. On the other hand, to address the role of “mother-derived factors”, in particular, hormones, we have used the standard protocol of pseudopregnancy, which simulates hormonal changes of real pregnancy in the absence of a fetus and thus excludes the influence of a fetus. Pseudopregnancy afforded the same protection from AKI and the same phenomenological changes as pregnancy did. Collectively, it leads to a conclusion that described protective effects are stimulated by hormonal changes in the maternal organism and apparently do not rely on fetus-derived factors. These findings are in agreement with previous studies^7,8^ demonstrating the same changes in liver and muscles regeneration in both normal pregnancy and pseudopregnancy. However, it has been shown that liver regeneration in pregnant animals is mostly due to hypertrophy rather than to hyperplasia^7^. In kidney, it seems that increased proliferation is a key factor, since real-time proliferation rates associated with higher PCNA and GDF11 expression was not accompanied by increased cell size after OGD in cell culture. It is not surprising, since liver and kidney cells are different in their capacity to alter cell size and tissue structure.

It is widely accepted that mitochondrial functioning associated with excessive reactive oxygen species production is of great importance during ischemic damage to organs, including kidney^10,51^. Herein we demonstrate that mitochondria isolated from pregnant animals have their energy functions less impaired by I/R, than in the control I/R group. The main difference between pregnant I/R and non-pregnant I/R groups is in the absence of low-energized renal mitochondria in the pregnant I/R group. It has been extensively discussed elsewhere^14,52^ that impaired (low-potential) mitochondria are the most crucial factor in acute pathology development. We also demonstrate that non-phosphorylated GSK3β, one of the key proteins, associated with a high probability of the mitochondria permeability transition pore opening and severity of ischemic damage, was much less expressed in cells obtained from pregnant animals. On the other hand, the protective, phosphorylated form of this kinase was much more abundant in the renal tissue of pregnancy. Note that the direct protective effect of GSK3β phosphorylation on AKI has been earlier shown^10,53^.

In conclusion, we want to address clinical experience that views pregnancy as a factor of risk and not of protection. As we have discussed earlier, in clinical practice, the total outcome of AKI is considered. However, as we have shown, it seems that this outcome is not determined by AKI severity. It is likely that overall outcome of AKI during pregnancy is greatly affected by complications unrelated to AKI *per se* that can happen during pregnancy, like septic abortion, puerperal sepsis, microangiopathy, endothelial dysfunction and preeclampsia^54^. We think that our data is important not only for fundamental science, but for clinical practice as well. Pregnancy makes kidneys more tolerant to acute kidney injury, and pseudopregnancy mimics these effects, which suggests that clinical strategies of AKI treatment during pregnancy might better focus more on complications, rather than on kidney damage itself.

## Methods

### Animals

Virgin female outbred rats of age 4–6 months were used. Previously, virgin rats were taken at 18^th^ day of pregnancy (third trimester), the day was checked both by the day of potential conceiving and manually by the size of embryos. Pseudopregnancy was induced by following protocol: virgin rats were injected with 5 IU human chorionic gonadotropin twice with 48 hours interval, after the second injection they were mated with vasectomized males. Pseudopregnant animals were taken at 7^th^ day after the first injection.

The animal protocols were evaluated and approved by the animal ethics committee of Belozersky Institute of Physico-Chemical Biology (Protocol 2/13 from April 8, 2013) in accordance with the Federation of Laboratory Animal Science Associations (FELASA) guidelines.

### I/R protocol

The animals anesthetized with chloralhydrate (300 mg/kg, i.p.) were subjected to 40-min warm ischemia of the left kidney as previously described^10^. The renal vascular bundle was occluded with a microvascular clip for 40 min. Circulation was restored by removing the clip. The lack of blood flow during ischemia and its restoring during reperfusion were assessed visually. Nephrectomy of the right kidney was executed simultaneously with ischemia of the left one. During operation, the body temperature of the rat was maintained at 37 ± 0.5 °C. Blood samples were taken 48 hours after ischemia from the tail to determine serum creatinine concentration using AU480 Chemistry System (Beckman Coulter, USA). On the first day after I/R, kidneys were taken for histopathological examination mainly for the presence of tubular necrosis. On the second day after the I/R, blood samples were taken, and kidneys were excised for the mitochondria isolation, determination of MDA in the tissue, western blotting. Kidney tissues were homogenized with a glass-teflon homogenizer in a PBS buffer containing 10 mM phenylmethylsulfonylfluoride at 4 °C. Two months after I/R, kidneys were examined for formation of fibrosis.

### Western blotting

Kidney homogenate or blood serum or cell culture samples were loaded onto 15% Tris-glycine polyacrylamide gels (10 μg protein/lane). After electrophoresis, gels were blotted onto PVDF membranes (Amersham Pharmacia Biotech, UK). Membranes were blocked with 5% non-fat milk in PBS/0.1% Tween-20 and subsequently incubated with primary antibodies to PCNA 1:1000 (ab13110, CellSignaling, USA), GDF11 1:500 (ab124721, Abcam, USA), NGAL (lipocalin 2) 1:1000 (ab63929, Abcam, USA), pGSK3β 1:1000 (9336, CellSignaling, USA), GSK3 α/β 1:1000 (sc7291, Santa Cruz Biotech, USA), VEGF 1:1000 (07–1376, Millipore, USA). Membranes were stained with secondary antibodies: anti-mouse IgG or anti-rabbit IgG conjugated with horseradish peroxidase 1:10000 (IMTEK, Russia). Detection was performed by V3 Western Blot Imager (BioRad, USA).

MDA concentration in kidney homogenate was determined using the thiobarbituric acid test as described in^55^.

### Erythropoietin immunoassay

Rats were placed in metabolic cages for the urine collection for 24 h. Collected urine was filtered and concentrated using the centrifugation with Amicon Ultra-4, 10 kDa (Millipore, USA) ultrafiltration units. The resulting retentates were assayed for erythropoietin level by ELISA Quantikine® assay according to the manufacturer’s instructions (R&D Systems, USA).

### MTT assay

Cell viability was evaluated by a widely used methyl thiazol tetrazolium (MTT) test. Cells in 96-well plates were incubated with MTT solution (2 mg/ml in DMEM/F12) for 120 min at 37 °C. Then the dye solution was removed, 50 µl DMSO per well was added, and the absorbance at 540 nm was measured by universal microplate reader Zenyth (Anthos Labtec, Austria).

### Renal histology

The kidney was isolated immediately after sacrificing the animal. It was then fixed in a 10% neutral buffered formalin solution, embedded in paraffin, and used for histopathological examination. Five-micrometer-thick sections were cut, deparaffinized, hydrated, and stained with hematoxylin/eosin or picro-fuchsin stain. Slices were examined for acute tubular necrosis in blinded fashion. For fibrosis measurements, the renal sections were examined in an automated fashion: fibrosis positive tissue percentage in the kidneys of all treated animals was estimated using ImageJ color threshold plugin. Fibrosis tissue was selected according to a specific color. Color threshold settings were adjusted manually for each image. Percentage of the field of view filled by fibrosis was considered to be fibrosis ratio. A minimum of seven fields of view for each kidney slice was examined.

### Primary adult rat kidney cells culture isolation

Tubular epithelial cells were isolated as previously described^56^. Briefly, kidneys from 4-month-old rats were excised under aseptic conditions. The tissue was manually shredded with scissors, washed 5 times in PBS from remaining blood and fat tissue and dissociated by collagenase treatment (0.5%, 30 min at 37 °C). The final suspension was centrifuged for 5 min at 50 × g. The pellet was resuspended in ∼10 mL of DMEM/F12 supplemented and kept for 2 min to discard large tissue pieces, after which the supernatant was transferred to another tube, and the pellet was repeatedly resuspended. After 10 min, the renal tubules were pelleted and dissociated. Single cells remaining in the suspension were discarded. The pellet was resuspended in DMEM/F12 with 10% FCS and EGF (10 ng/ml) and seeded onto special iCelligence 8-well plates and 96-well plates or onto 35-mm glass-bottom Petri dishes (FluoroDish, WPI, USA). The isolated culture was phenotyped with antibodies for Tamm-Horsfall glycoprotein, a kidney-specific protein produced by tubular cells. All cells in culture were positive for this marker (Suppl. 2).

### Real-time cell proliferation monitoring

Analysis of cells growth kinetics was performed using RTCA iCELLigence™ instrument (ACEA, San Diego, USA). This method is described in detail elsewhere^57^. In brief, the method is based on using electrical impedance of cell-covered electrodes. It was originally introduced in 1984^58,59^ and have been used for various studies of cell proliferation, viability, adhesion and migration^57,60,61^. The iCELLigence RTCA instrument was placed in a humidified incubator at 37 °C and 5% CO_2_. Cells were seeded on 8-well plates with microelectrodes; 24 hours after it, the medium was changed to dispose of unattached and dead cells. After next 24-hours, cells were subjected to 17 hours of OGD: medium was changed to Dulbecco’s phosphate-buffered saline (DPBS) and oxygen was replaced with N_2_ in multi-gas incubator Galaxy 170 R (Eppendorf/NewBrunswick, UK). After OGD the medium was changed back to the described above culture medium and normal O_2_ concentration.

### Mitochondria isolation

The rat kidney mitochondria were isolated by differential centrifugation^62^ in the medium containing 0.25 M sucrose, 10 mM Tris-HCl, 1 mM EDTA, 0.1% BSA, pH 7.4. Protein concentration was measured by bicinchoninic acid assay (Sigma, USA).

### Flow cytometry

Flow cytometry was performed using a Cytomics FC500 (Beckman Coulter, USA). Tetramethylrhodamine, ethyl ester (TMRE)-mediated fluorescence reflecting the magnitude of transmembrane potential was measured on the FL2-channel with λ_ex_ = 488 nm. The incubation medium contained 120 mM KCl, 3 mM HEPES, 1 mM EGTA, 5 mM K_2_PO_4_, 100 nM TMRE, 5 mM succinate, and 200 μg mitochondrial protein/ml, pH7.4. Data was analyzed with flowIT software and Microsoft Excel.

### Statistics

Values are presented as mean ± SEM. Comparisons between groups were made using Mann–Whitney U test. Data was analyzed in Microsoft Excel.

## Electronic supplementary material

Supplementary Information

## References

[1] Rewa O, Bagshaw SM (2014). Acute kidney injury—epidemiology, outcomes and economics. Nat. Rev. Nephrol..

[2] Machado S (2012). Acute kidney injury in pregnancy: a clinical challenge. J. Nephrol..

[3] Joseph KS (2010). Severe Maternal Morbidity in Canada, 2003 to 2007: Surveillance Using Routine Hospitalization Data and ICD-10CA Codes. J. Obstet. Gynaecol. Canada.

[4] Rao S, Jim B (2018). Acute Kidney Injury in Pregnancy: The Changing Landscape for the 21st Century. Kidney Int. reports.

[5] Mahesh E (2017). Pregnancy-related acute kidney injury: An analysis of 165 cases. Indian J. Nephrol..

[6] Popkov VA (2016). Molecular and Cellular Interactions between Mother and Fetus. Pregnancy as a Rejuvenating Factor. Biochemistry. (Mosc)..

[7] Gielchinsky Y (2010). Pregnancy restores the regenerative capacity of the aged liver via activation of an mTORC1-controlled hyperplasia/hypertrophy switch. Genes Dev..

[8] Falick Michaeli T (2015). The rejuvenating effect of pregnancy on muscle regeneration. Aging Cell.

[9] Gregg C (2007). White Matter Plasticity and Enhanced Remyelination in the Maternal CNS. J. Neurosci..

[10] Plotnikov EY (2007). The role of mitochondria in oxidative and nitrosative stress during ischemia/reperfusion in the rat kidney. Kidney Int..

[11] Zuk A, Bonventre JV (2016). Acute Kidney Injury. Annu. Rev. Med..

[12] Barrera-Chimal J (2012). Spironolactone prevents chronic kidney disease caused by ischemic acute kidney injury. Kidney Int..

[13] Goldberg MP, Choi DW (1993). Combined oxygen and glucose deprivation in cortical cell culture: calcium-dependent and calcium-independent mechanisms of neuronal injury. J. Neurosci..

[14] Zorov DB (2017). MitochondrialAging: Is There a Mitochondrial Clock?. J. Gerontol. A. Biol. Sci. Med. Sci..

[15] Cahill TJ, Choudhury RP, Riley PR (2017). Heart regeneration and repair after myocardial infarction: Translational opportunities for novel therapeutics. Nature Reviews Drug Discovery.

[16] Bruno S, Chiabotto G, Camussi G (2014). Concise Review: Different Mesenchymal Stromal/Stem Cell Populations Reside in the AdultKidney. Stem Cells Transl. Med..

[17] Plotnikov EY (2017). Intercellular Signalling Cross-Talk: To Kill, To Heal and To Rejuvenate. Heart. Lung Circ..

[18] Babenko VA (2015). Improving the Post-Stroke Therapeutic Potency of Mesenchymal Multipotent StromalCells by Cocultivation With Cortical Neurons: The Role of Crosstalk Between Cells. Stem Cells Transl. Med..

[19] Sinha M (2014). Restoring systemic GDF11 levels reverses age-related dysfunction in mouse skeletal muscle. Science.

[20] Olson KA (2015). Association of growth differentiation factor 11/8, putative anti-ageing factor, with cardiovascular outcomes and overall mortality in humans: analysis of the Heart and Soul and HUNT3 cohorts. Eur. Heart J..

[21] Loffredo FS (2013). Growth differentiation factor 11 is a circulating factor that reverses age-related cardiac hypertrophy. Cell.

[22] Plotnikov EY (2017). The role of oxidative stress in acute renal injury of newborn rats exposed to hypoxia and endotoxin. FEBS J..

[23] Silachev DN (2014). The Mitochondrion as a Key Regulator of Ischaemic Tolerance and Injury. Hear. Lung Circ..

[24] Juhaszova M (2009). Role of glycogen synthase kinase-3β in cardioprotection. Circulation Research.

[25] Van Wauwe J, Haefner B (2003). Glycogen synthase kinase-3 as drug target: from wallflower to center of attention. Drug News Perspect..

[26] Jope RS (2003). Lithium and GSK-3: One inhibitor, two inhibitory actions, multiple outcomes. Trends in Pharmacological Sciences.

[27] Bianchi DW, Zickwolf GK, Weil GJ, Sylvester S, DeMaria MA (1996). Male fetal progenitor cells persist in maternal blood for as long as 27 years postpartum. Proc. Natl. Acad. Sci. USA.

[28] Nassar D, Khosrotehrani K, Aractingi S (2012). Fetal microchimerism in skin wound healing. Chimerism.

[29] Kara RJ (2012). Fetal Cells Traffic to Injured Maternal Myocardium and Undergo Cardiac DifferentiationNovelty and Significance. Circ. Res..

[30] Zeng XX (2010). Pregnancy-associated progenitor cells differentiate and mature into neurons in the maternal brain. Stem Cells Dev..

[31] Wang Y (2004). Fetal cells in mother rats contribute to the remodeling of liver and kidney after injury. Biochem. Biophys. Res. Commun..

[32] Khosrotehrani K (2007). Fetal cells participate over time in the response to specific types of murine maternal hepatic injury. Hum. Reprod..

[33] Kleeberger W (2003). Increased chimerism of bronchial and alveolar epithelium in human lung allografts undergoing chronic injury. Am. J. Pathol..

[34] Feature S (2005). American Society of Nephrology Renal Research Report. J. Am. Soc. Nephrol..

[35] Pařízek A, Koucký M, Dušková M (2014). Progesterone, inflammation and preterm labor. J. Steroid Biochem. Mol. Biol..

[36] Szekeres-Bartho J, Polgar B (2010). PIBF: The Double Edged Sword. Pregnancy and Tumor. Am. J. Reprod. Immunol..

[37] Higgins DF (2007). Hypoxia promotes fibrogenesis *in vivo* via HIF-1 stimulation of epithelial-to-mesenchymal transition. J. Clin. Invest..

[38] Chang AS, Hathaway CK, Smithies O, Kakoki M (2015). Transforming growth factor β1 and diabetic nephropathy. Am. J. Physiol. - Ren. Physiol..

[39] Komala MG, Gross S, Zaky A, Pollock C, Panchapakesan U (2015). Linagliptin limits high glucose induced conversion of latent to active TGFβ through interaction with CIM6PR and limits renal tubulointerstitial fibronectin. PLoS One.

[40] Zhang Q (2010). *In vivo* delivery of gremlin siRNA plasmid reveals therapeutic potential against diabetic nephropathy by recovering bone morphogenetic protein-7. PLoS One.

[41] Kölling M (2017). Therapeutic miR-21 Silencing Ameliorates Diabetic Kidney Disease in Mice. Mol. Ther..

[42] Gomes SA, Hare JM, Rangel EB (2018). Kidney-Derived c-Kit ^+^ Cells Possess Regenerative Potential. Stem Cells Transl. Med..

[43] Schiessl IM (2018). Renal Interstitial Platelet-Derived Growth Factor Receptor-βCells Support Proximal Tubular Regeneration. J. Am. Soc. Nephrol..

[44] Conboy IM (2005). Rejuvenation of aged progenitor cells by exposure to a young systemic environment. Nature.

[45] Yuan L (2011). VEGF-modified human embryonic mesenchymal stem cell implantation enhances protection against cisplatin-induced acute kidney injury. Am. J. Physiol. Renal Physiol..

[46] Greenberg DA, Jin K (2013). Vascular endothelial growth factors (VEGFs) and stroke. Cell. Mol. Life Sci..

[47] Kanellis J, Mudge SJ, Fraser S, Katerelos M, Power DA (2000). Redistribution of cytoplasmic VEGF to the basolateral aspect of renal tubular cells in ischemia-reperfusion injury. Kidney Int..

[48] Jun JH, Jun NH, Shim JK, Shin EJ, Kwak YL (2014). Erythropoietin protects myocardium against ischemia-reperfusion injury under moderate hyperglycemia. Eur. J. Pharmacol..

[49] Sedaghat Z (2013). Remote preconditioning reduces oxidative stress, downregulates cyclo-oxygenase-2 expression and attenuates ischaemia-reperfusion-induced acute kidney injury. Clin. Exp. Pharmacol. Physiol..

[50] Ardalan, M. R. *et al*. Erythropoietin ameliorates oxidative stress and tissue injury following renal ischemia/reperfusion in rat kidney and lung. *Med. Princ. Pract*. **22** (2012). 10.1159/000340060 PMC5586709 23006583

[51] Ratliff BB, Abdulmahdi W, Pawar R, Wolin MS (2016). Oxidant Mechanisms in Renal Injury and Disease. Antioxid. Redox Signal..

[52] Banović MD, Ristić AD (2016). The Role of Mitochondrial Dysfunction in Heart Failure and Potential Therapeutic Targets. Curr. Pharm. Des..

[53] Hu B (2016). GSK-3beta Inhibitor Induces Expression of Nrf2/TrxR2 Signaling Pathway to Protect against Renal Ischemia/Reperfusion Injury in Diabetic Rats. Kidney Blood Press. Res..

[54] Acharya A, Santos J, Linde B, Anis K (2013). Acute Kidney Injury in Pregnancy—Current Status. Adv. Chronic Kidney Dis..

[55] Mihara M, Uchiyama M (1978). Determination of malonaldehyde precursor in tissues by thiobarbituric acid test. Anal. Biochem..

[56] Van der Hauwaert C (2013). Isolation and characterization of a primary proximal tubular epithelial cell model from human kidney by CD10/CD13 double labeling. PLoS One.

[57] Atienza JM (2006). Dynamic and label-free cell-based assays using the real-time cell electronic sensing system. Assay Drug Dev. Technol..

[58] Giaever I, Keese CR (1984). Monitoring fibroblast behavior in tissue culture with an applied electric field. Proc. Natl. Acad. Sci. USA.

[59] Giaever I, Keese CR (1991). Micromotion of mammalian cells measured electrically. Proc. Natl. Acad. Sci. USA.

[60] Atienza JM, Zhu J, Wang X, Xu X, Abassi Y (2005). Dynamic Monitoring of Cell Adhesion and Spreading on Microelectronic Sensor Arrays. J. Biomol. Screen..

[61] Wegener J, Keese CR, Giaever I (2000). Electric Cell–Substrate Impedance Sensing (ECIS) as a Noninvasive Means to Monitor the Kinetics of Cell Spreading to Artificial Surfaces. Exp. Cell Res..

[62] Johnson D, Lardy H (1967). Isolation of liver or kidney mitochondria. Methods Enzymol..

