# Supplementary material for: Pregnancy protects the kidney from acute ischemic injury
Source: Sci Rep. 2018 Sep 28;8:14534. doi: 10.1038/s41598-018-32801-8 (PMC6162317; doi:10.1038/s41598-018-32801-8)
Supplement: Supplementary file 1 — Supplementary Information [file 41598_2018_32801_MOESM1_ESM.pdf]

Pregnancy protects the kidney from acute ischemic injury

Vasily A. Popkov[1,2,3], Nadezda V. Andrianova[2], Vasily N. Manskikh[1], Denis N. Silachev[1,3], Irina B. Pevzner[1,3], Ljubava D. Zorova[1,3], Gennady T. Sukhikh[3], Egor Y. Plotnikov[1,3,4] & Dmitry B. Zorov[1,3]

[1]Belozersky Institute of Physico-Chemical Biology, Lomonosov Moscow State University, Moscow, Russia

[2]Faculty of Bioengineering and Bioinformatics, Lomonosov Moscow State University, Moscow, Russia

[3]V.I. Kulakov National Medical Research Center of Obstetrics, Gynecology and Perinatology, Moscow, Russia

[4]Institute of Molecular Medicine, Sechenov First Moscow State Medical University, Moscow, Russia

*Correspondence and requests for materials should be addressed to E.Y.P. and D.B.Z.

(e-mails: plotnikov@belozersky.msu.ru, zorov@belozersky.msu.ru)

SUPPLEMENTAL METHODS

Real-time PCR. To detect the presence of a fetus-derived cells in kidney tissue of pregnant animals real-time PCR with primers for Y-chromosome was used. RT-PCR reactions were carried out in an one-tube format on the Bio-Rad CFX96 platform using one-step RT-PCR kit (alpha-ferment) according to the manufacturer's protocol. A total RNA purified from whole kidney tissue with RNeasy Mini Kit (QIAGEN) served as a template in combined RT and PCR reactions. Relative TSPY1 (testis specific protein, Y-linked 1) expression was measured in TaqMan Gene Expression. Thermal settings for the RT-PCR reaction were: one cycle of reverse transcription at 50 C for 15 min followed by one cycle of Taq-AT enzyme activation at 95 C for 5 min, then 45 cycles of 15 sec denaturation at 95 C, 15 sec annealing at 50 C and 10 sec extension at 72 C with fluorescent signal acquisition.
Primers and probes used for TSPY1amplification:

GTGAAGCCTCTGGTAATATGTG	TSPY1F
TCTACCACCTCTGTGACTTTC	TSPY1R
6FAM-TCCGCCATAGTAGCCTCGTCACC-BHQ1	TSPY1TM

Confocal-microcopy. We have also checked the presence of fetus-derived cells using confocal microscopy. For this purpose, GFP-transgenic males were bred with wild-type females, resulting in some fetus being GFP-transgenic as well (Suppl. 1B). We were able to detect single GFP-positive cells in vital slices of mothers kidney, confirming the presence of such cells in mothers tissue (Suppl.1C). Images were obtained using a LSM510 inverted confocal microscope (Carl Zeiss, Jena, Germany), slices were made using Vibroslice vibrating microtome (WPI, Sarasota, FL, USA), and placed in 35-mm glass-bottom Petri dishes in DMEM/F12 culture medium.

Primary kidney tubular epithelial cells phenotyping was performed using anti-Tamm-Horsfall glycoprotein antibodies (1:200, AB733, Chemicon, USA), which is a kidney-specific protein produced by tubular cells. Images were obtained using a LSM510 inverted confocal microscope (Carl Zeiss, Jena, Germany).

SUPPLEMENTAL FIGURES LEGENDS

FIGURE 1. Presence of microchimeric fetal cells in mother's kidney. (A) Real-time PCR with primers for Y-chromosome. Male kidney has the noticeable presence of Y-chromosome in tissue, while there is no PCR-product in female tissue. In the pregnant female kidneys, there is a product on later cycles, indicating the presence of Y-chromosome carrying cells in female kidney-tissue, which can only originate from male fetus(es), (B) Fluorescent imaging of GFP-positive embryo in GFP-negative mother (C) Confocal microscopy, showing presence of a GFP-positive cell, originated from GFP-transgenic fetus, in the kidney tissue slice of wild-type mother.

FIGURE 2. Primary kidney tubular epithelial cells phenotyping. (A) Microscopy of the kidney tubules during isolation process; (B) Phase contrast image of the primary tubular epithelial cells two days after *in vitro* cultivation; (C) Fluorescent microscopy of the same cells labeled with anti-Tamm-Horsfall glycoprotein antibodies.

SUPPLEMENTAL FIGURE 1

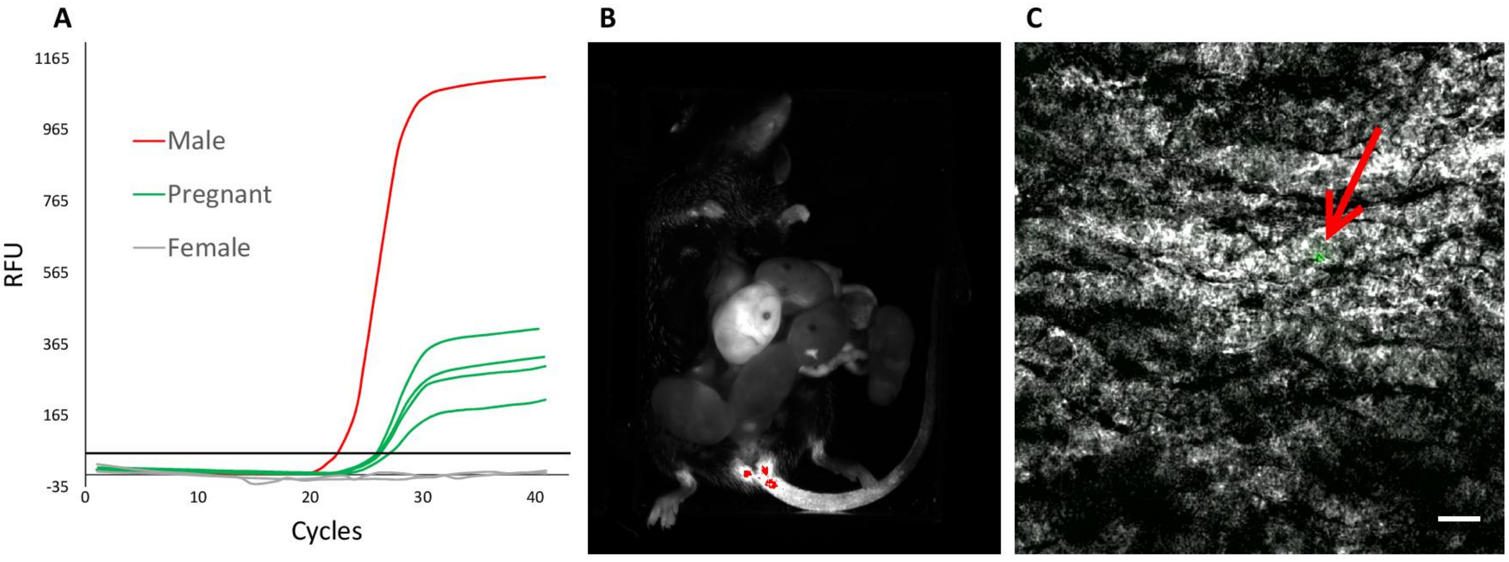

SUPPLEMENTAL FIGURE 2

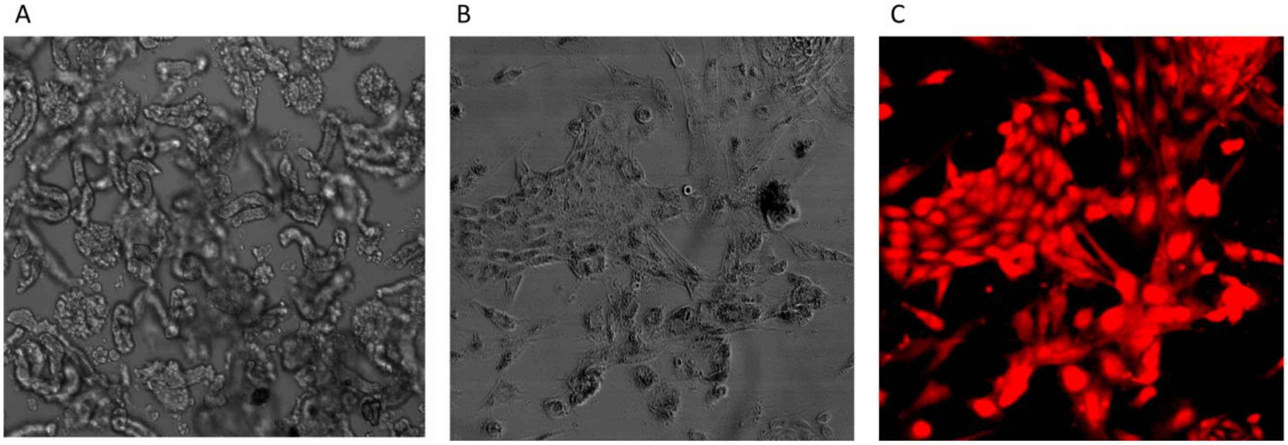

Full-length blots for main text figures

Figure 1B

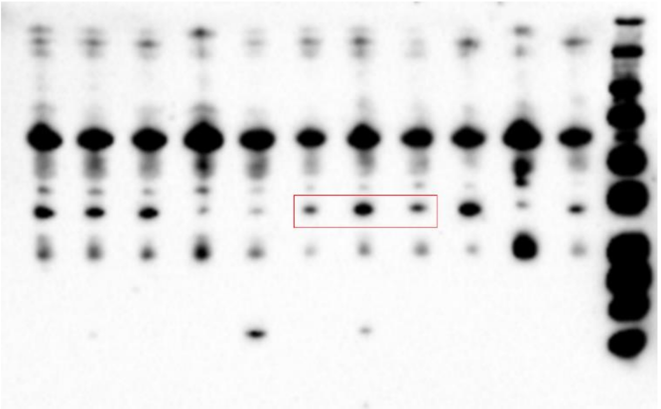

Figure 5A

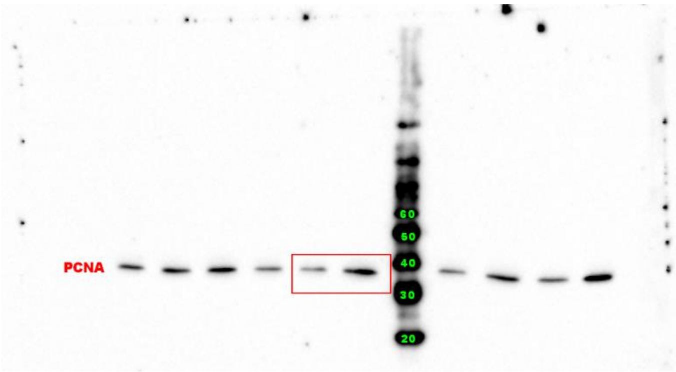

Figure 5B

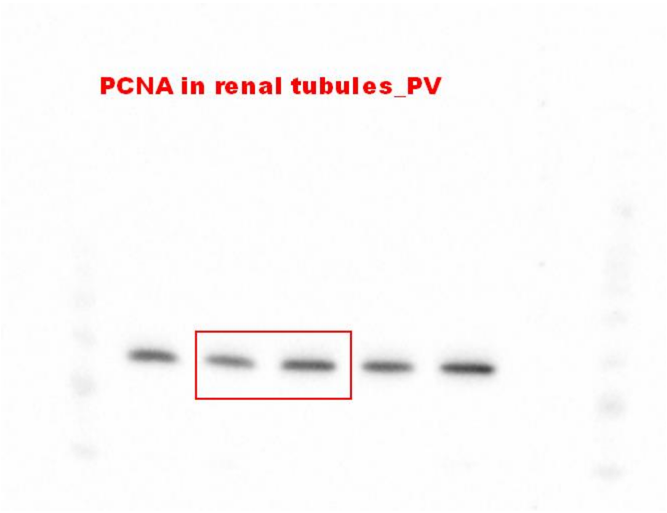

Figure 5C

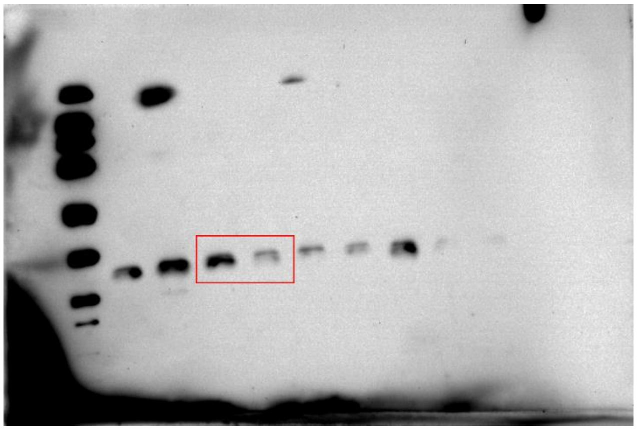

Figure 5D

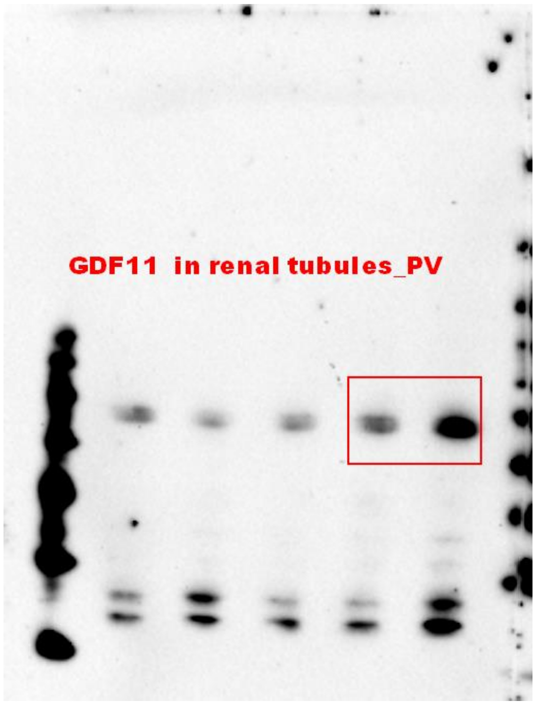

Figure 5F

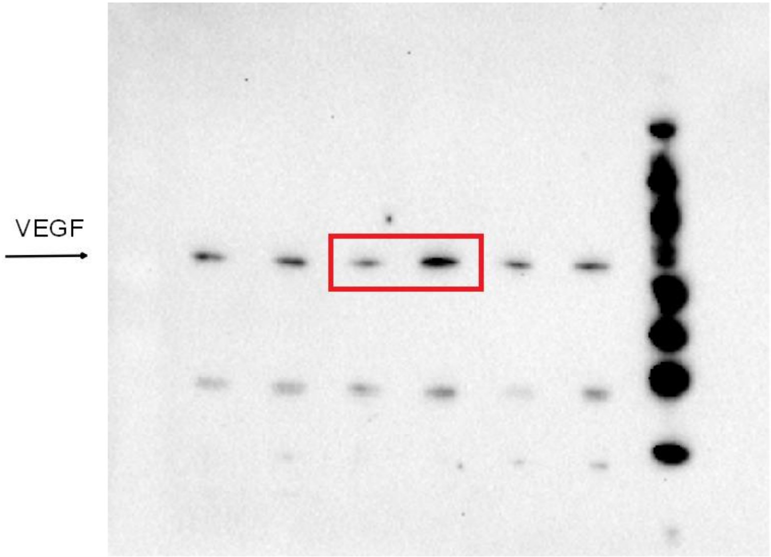

Figure 6A

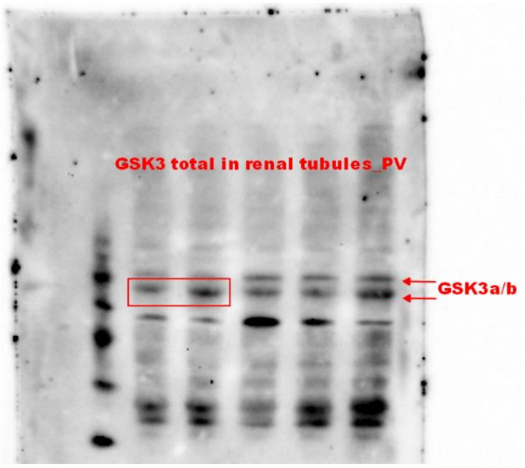

Figure 6B

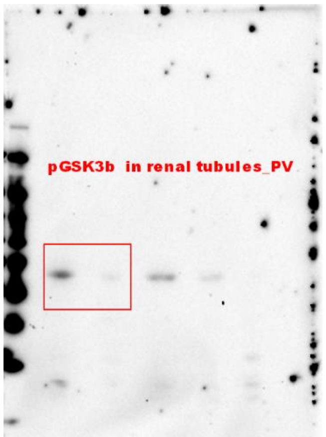
pGSK3b in renal tubules_PV
